# Crystallinity and Sub-Band Gap Absorption of Femtosecond-Laser Hyperdoped Silicon Formed in Different N-Containing Gas Mixtures

**DOI:** 10.3390/ma10040351

**Published:** 2017-03-28

**Authors:** Haibin Sun, Jiamin Xiao, Suwan Zhu, Yue Hu, Guojin Feng, Jun Zhuang, Li Zhao

**Affiliations:** 1 Collaborative Innovation Center of Advanced Microstructures, State Key Laboratory of Surface Physics and Department of Physics, Fudan University, Shanghai 200433, China; sunhaibin007@gmail.com (H.S.); 14110190075@fudan.edu.cn (S.Z.); 2 Department of Optical Science and Engineering, Fudan University, Shanghai 200433, China; 12110190077@fudan.edu.cn (J.X.); 14110720002@fudan.edu.cn (Y.H.); 3 Spectrophotometry Laboratory, National Institute of Metrology, Beijing 100013, China; fengguojin@nim.ac.cn

**Keywords:** femtosecond laser, hyperdoped, nitrogen, crystallinity, sub-band gap absorption

## Abstract

Femtosecond (fs)-laser hyperdoped silicon has aroused great interest for applications in infrared photodetectors due to its special properties. Crystallinity and optical absorption influenced by co-hyperdoped nitrogen in surface microstructured silicon, prepared by fs-laser irradiation in gas mixture of SF_6_/NF_3_ and SF_6_/N_2_ were investigated. In both gas mixtures, nitrogen and sulfur were incorporated at average concentrations above 10^19^ atoms/cm^3^ in the 20–400 nm surface layer. Different crystallinity and optical absorption properties were observed for samples microstructured in the two gas mixtures. For samples prepared in SF_6_/N_2_, crystallinity and light absorption properties were similar to samples formed in SF_6_. Significant differences were observed amongst samples formed in SF_6_/NF_3_, which possess higher crystallinity and strong sub-band gap absorption. The differing crystallinity and light absorption rates between the two types of nitrogen co-hyperdoped silicon were attributed to different nitrogen configurations in the doped layer. This was induced by fs-laser irradiating silicon in the two N-containing gas mixtures.

## 1. Introduction

Hyperdoped silicon prepared by fs-laser irradiation exhibits a spiked surface and supersaturated dopants in the doped layer [1,2,3,4,5,6]. This lowers the optical reflectance of the surface and increases sub-band gap absorptance coefficient [7,8,9,10,11,12]. Therefore, femtosecond (fs)-laser hyperdoped silicon shows strong broadband light absorptance, with potential applications for photovoltaics and infrared photodetectors [13,14,15,16,17]. In fact, infrared photolectronic response has been observed in plain gold-hyperdoped silicon and spiked sulfur-hyperdoped silicon [15,17,18]. Before hyperdoped silicon is used for photoelectric devices, the crystallinity in the doped layer damaged by fs-laser irradiation should be recovered by thermal annealing. This causes significant sub-band gap absorption declines [6,19,20,21,22]. The post-processing method of nanosecond (ns)-laser melting after fs-laser ablation was reported by Franta et al. to simultaneously maintain high crystallinity and strong sub-band gap absorption [23].

Besides post-processing methods, high crystallinity is achieved in hyperdoped silicon during the process of preparation by ion implantation [24]. In our previous work, a method of fs-laser co-doping technique in SF_6_/NF_3_ used to obtain hyperdoped silicon showed the ability to simultaneously possess high crystallinity and strong sub-band gap absorption by co-hyperdoping with nitrogen and sulfur [25]. The co-hyperdoped nitrogen from SF_6_/NF_3_ is deemed to effectively improve the crystallinity by repairing defects in the doped layer [26,27,28,29,30,31,32]. Based on this result, whether other N-containing gas (e.g., N_2_) can also introduce similar effects on crystallinity raises potential future research questions. If N_2_ does offer positive outcomes, this will create additional choices for N-containing gas used in the fs-laser co-hyperdoping method; even if outcomes are not favorable, the different effects from NF_3_ will help researchers to understand the mechanisms for obtaining improved crystallinity via a nitrogen co-doping method. This work presents recent progress on this issue. Hyperdoped silicon was fabricated by fs-laser irradiation in SF_6_/N_2_ gas-mixtures, and its crystallinity and light absorption properties were investigated. Although super-statured nitrogen and sulfur are also incorporated into the doped layer for gas mixtures of SF_6_/N_2_, different crystallinity and optical absorption rates were observed for samples prepared in gas mixtures of SF_6_/NF_3_ and SF_6_/N_2_. The configurations of nitrogen doped in silicon introduced by the fs-laser irradiation in the two N-containing gas mixtures, and their different effects on the crystallinity and light absorption is discussed.

## 2. Materials and Methods

Silicon wafers (*p* (100), ρ = 1–3 Ω/cm^2^, *d* = 250 ± 10 μm) were cleaned to remove organic and metallic contaminants by the Radio Corporation of America (RCA) standard process. The cleaned Silicon wafers were placed in a stainless-steel vacuum chamber and irradiated by a Yb:KGW fs-laser (515 nm, 190 fs, and 1 kHz) at normal incidence. The chamber was filled with gas mixture of SF_6_/NF_3_ or SF_6_/N_2_ at 70 kPa. The laser beam was focused to a spot size of 60 μm in diameter on the samples with a 250-mm focal length lens. The silicon wafer was mechanically translated by stepper motors, and the areas of the samples could be achieved at 10 × 10 mm^2^. The stepper motors translated at a speed of 500 μm/s, and the scanning line interval chose the radius of the laser spot (about 30 μm). In this way, any given spot in the irradiated region was exposed to about 350 laser pulses.

The morphology of the textured surface of the fs-laser hyperdoped silicon was observed by a scanning electron microscope (SEM, Hitachi, Tokyo, Japan). The average concentrations of the doped nitrogen and sulfur in the surface layer were detected by secondary ion mass spectrometry (SIMS, Xevo TQD, SCIEX, Framingham, MA, USA) measurements. The crystal properties of samples were examined by a confocal Raman spectroscopy (excited by a He-Ne laser of 633 nm, Horiba Jobin Yvon XploRA, Paris, France). The structures of nitrogen in silicon lattices were explored by a Fourier transform infrared (FTIR) spectrometer (Nicolet Nexus 470, Nicolet Nexus, Madison, WI, USA). Finally, using a spectrophotometer (Varian Cary 5E UV-VIS-NIR, Varian Cary, Palo Alto, CA, USA) equipped with an integrating sphere, we collected the transmission (*T*) and reflection (*R*) spectra of samples in the wavelength of 250–2500 nm, respectively, and determined the absorptance (*A*) by *A* = 1 − *R* − *T*.

## 3. Results and Discussion

### 3.1. Surface Morphology

**Figure 1:**
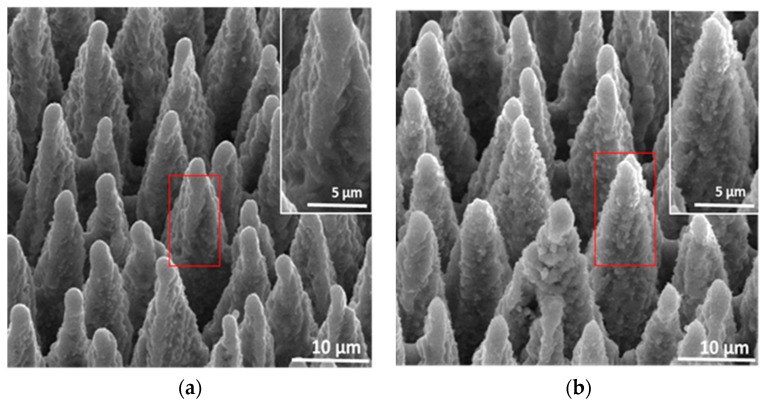
SEM images (viewed at 45°) of the fs-laser hyperdoped silicon formed in gas mixture of (**a**) SF_6_/NF_3_ and (**b**) SF_6_/N_2_ at laser fluence of 12.1 kJ/m^2^. Both gas mixtures are composed by partial pressure ratio of 35:35 kPa.

As in SF_6_/NF_3_, fs-laser irradiation in a gas mixture of SF_6_/N_2_ also induces arrays of sharp spikes in the surface of silicon, as shown in Figure 1. While the spikes formed in a gas mixture of SF_6_/NF_3_ have a relatively smoother surface (Figure 1a), those prepared in SF_6_/N_2_ exhibit a rough surface with overlying numbers of nm-granular structures (Figure 1b). The difference in the degree of spike surface roughness is attributed to different effects of NF_3_ and N_2_ during the fs-laser fabrication processes [26].

### 3.2. Nitrogen Co-Doping Characters

Similar to that in SF_6_/NF_3_ [25], the hyperdoped silicon formed in SF_6_/N_2_ also co-doped with supersaturated sulfur and nitrogen in the surface layer. The average doping concentrations of sulfur and nitrogen over a depth range of 20–400 nm in the surface layer are shown in Table 1. Compared with the average doping concentration of nitrogen in the silicon materials prepared in SF_6_/NF_3_, those introduced by SF_6_/N_2_ are slightly lower. The lower doping concentration exceeded 10^19^ atoms/cm^3^ of the surface layer, which is several orders of magnitude above its solid solubility in silicon crystals [26,30]. Additionally, the co-doping concentrations of sulfur induced by the two gas mixtures are very similar. The difference of the doped nitrogen induced by the two N-containing gas mixtures was determined by the doping method. This will also affect the crystallinity of the doped layer.

### 3.3. Crystal Properties

**Figure 2:**
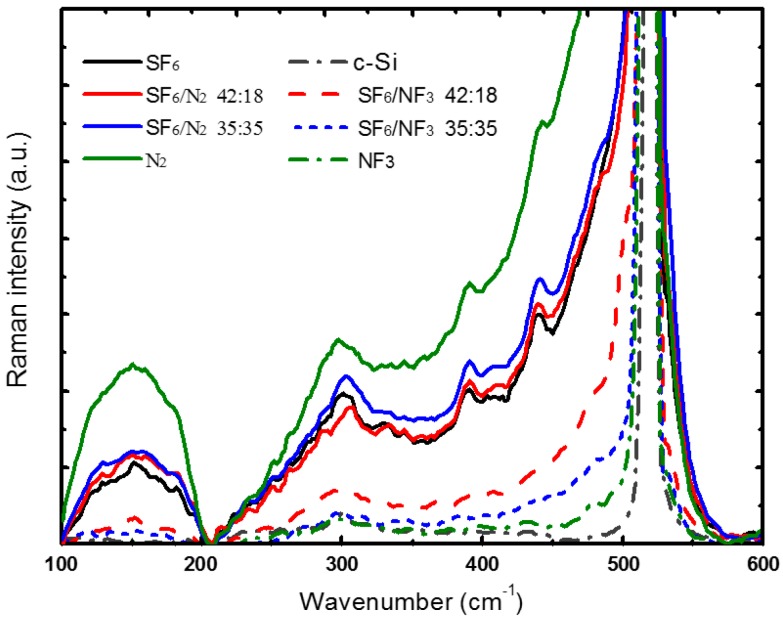
Raman spectra of hyperdoped silicon prepared in gas mixture of SF_6_/NF_3_ and SF_6_/N_2_ at respective ratios. All the samples formed at same laser fluence of 12.1 kJ/m^2^, and the crystalline silicon is given as comparison.

The crystallinity of the laser hyperdoped silicon influenced by supersaturated nitrogen from SF_6_/NF_3_ and SF_6_/N_2_ were detected by Raman diagnosis. The results are shown in Figure 2. Results show normalized Raman spectra of hyperdoped silicon prepared in gas mixture of SF_6_/NF_3_ and SF_6_/N_2_ at different pressure ratios. Compared with sample prepared in SF_6_, an improved crystallinity with a small amount of amorphous silicon and little polymorph silicon was obtained for samples formed in SF_6_/NF_3_. Hyperdoped silicon prepared in SF_6_/N_2_ showed that the crystallinity in the doped layer was little improved by the co-hyperdoped nitrogen. As the pressure ratio of N_2_ in SF_6_/N_2_ increased, that of SF_6_ decreased. The Raman peaks assigned to polymorphic BC8 structure (Si-III, 387 cm^−1^ and 443 cm^−1^) [20,21,25,26] remained nearly unchanged, and the amorphous silicon (a-Si, broad peaks at 300 cm^−1^ and 460–495 cm^−1^) [20,21,25,26] increased. Furthermore, as the pressure ratio of N_2_ increased, the width of Raman peaks at 520 cm^−1^ also increased, which implies that the lattice stress increased and/or the grain size decreased [23]. Therefore, we can draw the conclusion that the hyperdoped nitrogen introduced by gas mixtures of SF_6_/NF_3_ and SF_6_/N_2_ show different effects on the crystallinity in the doped layer.

As reported, nitrogen can be doped in silicon lattices in different configurations, such as single nitrogen atoms, paired nitrogen atoms, and nitrogen molecules [26,31,32,33]. With the irradiation of fs-laser pulses, nitrogen atoms were dissociated from NF_3_ and then doped in silicon lattices at a substitutional or interstitial site (single atoms and paired atoms). The super-statured nitrogen atoms in the doped layer combine with vacancies to form various nitrogen vacancies complexes [26,32,33]. These complexes effectively improved the crystallinity of the hyperdoped silicon by locking dislocations and suppressing the formation of large defects [25,26,29,30]. Although the gas mixture of SF_6_/N_2_ also introduced supersaturated nitrogen in the doped layer, this hyperdoped nitrogen exists in silicon lattices as other nitrogen configurations. As Takao reported, during the process of silicon growth in N_2_, nitrogen molecules doped in silicon showed minimal interaction with silicon lattices [34]. Even with fs-laser irradiation, the N_2_ could not be dissociated into single nitrogen atoms and doped in silicon. This is attributed to the laser fluence of 12.1 kJ/m^2^ (approximately 10^7^ W/m^2^), far from the ionization threshold of N_2_ (≥10^14−15^ W/m^2^) [35,36].

**Figure 3:**
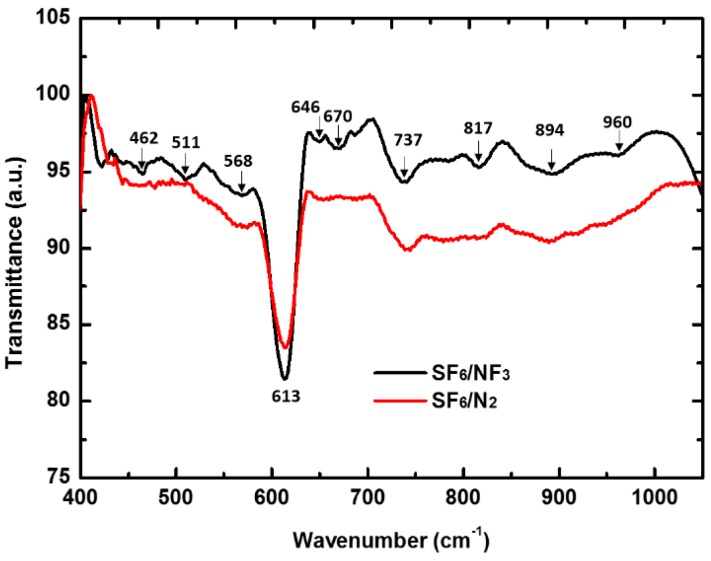
FTIR spectra of the nitrogen co-hyperdoped silicon prepared in SF_6_/NF_3_ and SF_6_/N_2_. Both gas mixtures are composed by a partial pressure ratio of 35:35 kPa.

To further understand the structure of the co-hyperdoped nitrogen in silicon introduced by fs-laser irradiation in SF_6_/NF_3_ and SF_6_/N_2_, IR detection was performed on the samples using a Fourier transform infrared (FTIR) spectrometer. In the results shown in Figure 3, the spectra of samples prepared in SF_6_/NF_3_, showed several IR bands at 462, 511, 568, 613, 646, 670, 737, 817, 894, and 960 cm^−1^. For the spectra of samples prepared in SF_6_/N_2_, there were only two obvious IR bands at 613 and 737 cm^−1^. According to calculations, the IR band at 960 cm^−1^ is assigned to a nitrogen di-interstitial pair (N_i_–N_i_); the IR bands at 568, 646, and 670 cm^−1^ are assigned to local vibrational modes of (N_i_–N_i_) self-interstitial complexes sub-situational nitrogen pairs (Ns–Ns), (N_i_–N_i_) vacancy complexes, and (Ns–Ns) vacancy complexes, respectively [23,26,32]. The IR bands at 613 and 737 cm^−1^ are connected with crystalline silicon [23,32]. All of the IR bands assigned to nitrogen-containing complexes (568, 646, and 670 cm^−1^) were unobserved in the spectra of samples prepared in SF_6_/N_2_. The FTIR spectra measurement more clearly show that super-saturated nitrogen from SF_6_/NF_3_ and SF_6_/N_2_ doped as different configurations in silicon lattices, and show different effects on defects in the doped layer. Similar to effects on defects, nitrogen configurations also exhibit different effects on the light absorption of the fs-laser hyperdoped silicon.

### 3.4. Optical Light Absorption

**Figure 4:**
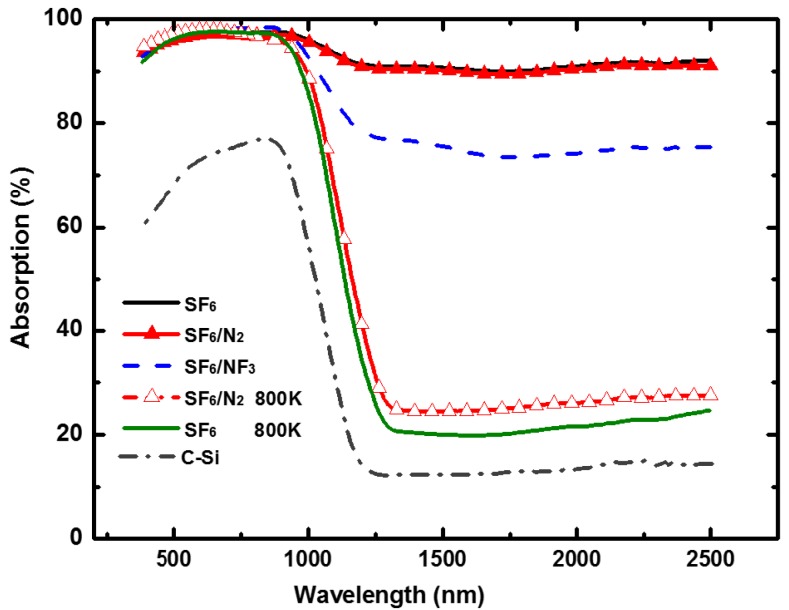
Absorption of the hyperdoped silicon prepared in SF_6_, SF_6_/NF_3_, SF_6_/N_2_. That of the annealed (800 K, 30 min) samples and crystalline silicon are given as comparison.

Figure 4 shows the light absorption properties of nitrogen co-hyperdoped silicon prepared in SF_6_/NF_3_ (35:35 kPa) and SF_6_/N_2_ (35:35 kPa). Compared with the sub-band gap absorption of the silicon materials prepared in SF_6_, that of the sample formed in SF_6_/NF_3_ slightly decreased, but it was nearly unchanged (keeps at 90%) for the silicon materials prepared in SF_6_/N_2_. As we noted above, the hyperdoped silicon formed in SF_6_/NF_3_ showed higher crystallinity, and the doped nitrogen from SF_6_/N_2_ failed to improve crystallinity in the doped layer (see Figure 2b). Samples prepared in SF_6_/N_2_ should be annealed in order to recover crystallinity before use in photoelectric devices. Unfortunately, after annealing the samples under a flash lamp at 800 K for 30 min in forming gas (95% N_2_, 5% H_2_, 300 sccm), the sub-band gap absorption of the samples prepared in SF_6_/N_2_ declined to approximately 25%. This light absorption property is nearly identical to that of samples prepared in SF_6_, which shows a strong sub-band gap absorption (more than 90%) before annealing, and declines sharply (to 20%–30%) after annealing. Sulfur co-hyperdoped silicon remains a method that can induce stronger sub-band gap absorption. The light absorption properties were determined by the doping method.

According to previous research [25,33], similar to sulfur atoms, the hyperdoped single nitrogen atoms in silicon could import defect states into the silicon gap and induce sub-band gap absorption. The hyperdoped paired nitrogen atoms induced no defect states and demonstrate few contributions to sub-band gap absorption. Additionally, the sub-band gap absorption induced by hyperdoped single nitrogen atoms is lower than that induced by hyperdoped sulfur, which made the sub-band gap absorption of samples prepared in SF_6_/NF_3_ decline slightly [25,33]. For samples prepared in SF_6_/N_2_, supersaturated nitrogen molecules were induced to co-dope in the surface layer, which exhibit few interactions with silicon lattices [34]. The sub-band gap absorption of samples prepared in SF_6_/N_2_ was nearly unchanged by the hyperdoped nitrogen molecules, dependent on the co-hyperdoped sulfur atoms in the doped layer. Samples were prepared at several pressure ratios of SF_6_/N_2_, and found that all show a sub-band gap absorption similar to those prepared in SF_6_. The differences of the mechanism on the sub-band gap absorption induced by sulfur atoms and that of those incorporated with nitrogen molecules doping in silicon requires further study.

## 4. Conclusions

This study examined different configurations of nitrogen doped in silicon, introduced by N-containing gas of SF_6_/NF_3_ and SF_6_/N_2_, and the associated effects on crystallinity and sub-band gap absorption of fs-laser hyperdoped silicon. Although supersaturated nitrogen and sulfur are incorporated into the doped layer for both gas mixtures, the co-hyperdoped nitrogen from SF_6_/NF_3_ improved the crystallinity, while samples processing in SF_6_/N_2_ showed almost no effects on both crystallinity and sub-band gap absorption. This is because the supersaturated nitrogen molecules induced by SF_6_/N_2_ in the doped layer showing little interaction with the silicon lattice. This is largely different from that of induction by SF_6_/NF_3_, which induced supersaturated nitrogen atoms doped in silicon and formed various nitrogen vacancy complexes to improve the crystallinity of the doped layer. The doped nitrogen molecules from SF_6_/N_2_ failed to improve crystallinity in hyperdoped silicon; as a comparison, it makes the mechanism and demands for obtaining the improved crystallinity by such nitrogen co-doping technique increasingly clear. The investigations of the effects of nitrogen configuration on the crystallinity and light absorption of hyperdoped silicon will greatly benefit non-equilibrium material manufacturing.

## Figures and Tables

**Table 1:** Average concentration of nitrogen and sulfur in the surface layer (20–400 nm depth) of hyperdoped silicon formed in SF_6_/NF_3_ and SF_6_/N_2_.

Pressure Ratio of Gas Mixture	N (10^19^ Atoms/cm^3^)	S (10^19^ Atoms/cm^3^)
SF_6_/NF_3_ (35:35 kPa)	4.50298	4.43861
SF_6_/N_2_ (35:35 kPa)	1.31992	4.56782
